# Activation of Ocular Syphilis After Small-Incision Lenticule Extraction

**DOI:** 10.7759/cureus.32299

**Published:** 2022-12-07

**Authors:** Stephen A LoBue, Thomas Catapano, Brittany B DeNaro, Christopher Shelby, Wyche T Coleman

**Affiliations:** 1 Department of Ophthalmology, Willis-Knighton, Shreveport, USA; 2 Department of Ophthalmology, St. George’s University School of Medicine, West Indies, GRD

**Keywords:** neurosyphilis, monovision, smile, anterior uveitis, ocular syphilis

## Abstract

A 41-year-old female presented to the Willis-Knighton Eye Institute to undergo evaluation for refractive surgery. The patient had a best-corrected visual acuity for a distance of 20/15-1 of the right eye (OD) and 20/15-1 of the left eye (OS) with a manifest refraction of -2.75 OD and -1.75 OS. Near visual acuity was J1+ in both eyes (OU). A trial of a monovision contact lens was successful with the dominant eye selected for distance. The patient was then planned for small-incision lenticule extraction (SMILE) OD only with a plano target. SMILE was performed and was uncomplicated with uncorrected visual acuity of 20/15- on postoperative day one. Two weeks after the initial SMILE procedure, the anterior segment was notable for 1-2+ cells OD. Topical prednisone was changed to difluprednate 0.05% TID OD with improvement in symptoms. However, the anterior chamber cell was never fully resolved by month three. A systemic workup revealed a positive rapid plasma reagin with 1:64 titer and a positive fluorescent treponemal antibody absorption in a patient never treated for syphilis. The patient was diagnosed with ocular syphilis and received a two-week course of intravenous penicillin G. A slow topical prednisone tapper was performed with the resolution of inflammation by one year. Anterior uveitis after refractive surgery is uncommon. The incidence of anterior uveitis after SMILE is even rarer with no previously documented incidence in the literature. As a result, persistent cell seen in refractive procedures, especially SMILE, is a concerning finding, warranting further workup to rule out underlying systemic diseases including syphilis.

## Introduction

Laser refractive surgery has been performed for more than 30 years and is considered to be one of the safest and most efficacious treatments for moderate refractive errors [1].

Advancements in laser refractive surgery have led to the development of small-incision lenticule extraction (SMILE), a flapless femtosecond laser‑assisted refractive procedure that can treat a variety of refractive errors. SMILE offers the advantage of increased biomechanical strength of remaining tissue with reduced incidence of dry eye disease [2].

Although SMILE avoids the flap‑related complications associated with laser‑assisted in‑situ keratomileusis (LASIK), the procedure is technically more challenging with a host of possible intraoperative complications, including loss of suction, the formation of the altered opaque bubble layer (OBL), difficulty in lenticular dissection or extraction, cap perforation, and decentered ablation [3].

Various postoperative problems have been documented, including interface debris, epithelial ingrowth, interface fluid syndrome, pressure‑induced stromal keratitis, infectious keratitis, diffuse lamellar keratitis, transient light sensitivity syndrome (TLSS), and dry eye [4].

The development of anterior uveitis after LASIK is rare and has not been adequately documented with regard to SMILE [4]. To our knowledge, we present the first possible case of anterior uveitis secondary to latent syphilis after a SMILE procedure.

## Case presentation

A 41-year-old female with no medical history presented to the Willis-Knighton Eye Institute to undergo evaluation for refractive surgery. As a low myope, the patient had adequate uncorrected near visual acuity (UNVA) but requested spectacle independence for distance. The patient also complained of severe dry eyes not improved with artificial tears with a familial ocular history positive for glaucoma (father) and Sjogren’s syndrome (mother). The patient had a best-corrected visual acuity (BCVA) for a distance of 20/15-1 of the right eye (OD) and 20/15-1 of the left eye (OS) with a manifest refraction of -2.75 OD and -1.75 OS. UNVA was J1+ in both eyes (OU).

Slit lamp examination demonstrated a clear cornea with deep anterior chambers without signs of inflammation OU. Trace nuclear sclerosis was also noted with a normal fundus examination.

**Figure 1:**
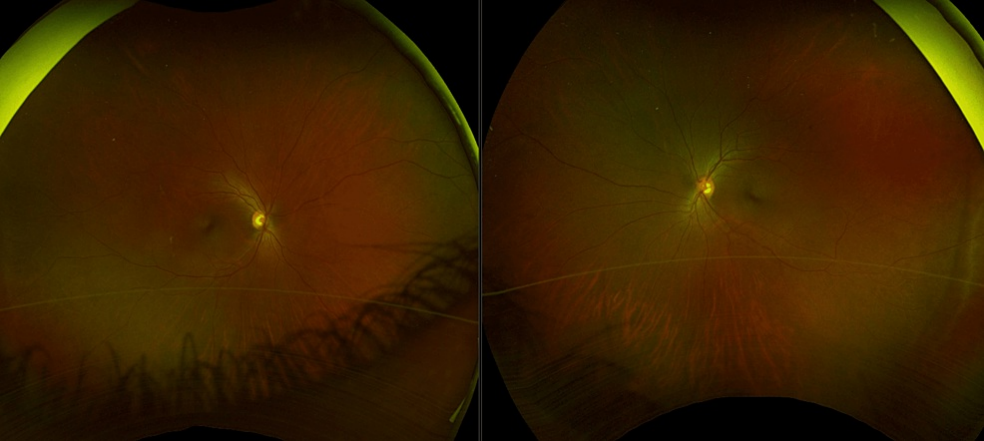
Optos ultra-widefield retinal imaging of both eyes. A clear media was noted without the presence of vitritis in both eyes. The optic nerve was well perfused with a cup-to-disc ratio of 0.5. Normal caliber and course of the vessels were noted without vasculitis. The macula and surrounding retina were clear without any signs of retinal or choroidal lesions in both eyes.

The intraocular pressure (IOP) was 12 mmHg OD and 13 mmHg OS with a central corneal thickness (CCT) of 607 OD and 599 OS.

The patient was started on cyclosporin 0.05% BID OU. She was evaluated four months later with a resolution of dry eye symptoms and continued interest in refractive surgery. A trial of monovision contact lens was initiated with the dominant right eye corrected to plano with the left eye uncorrected for near. At the two-week follow-up, the patient adequately tolerated monovision with BCVA 20/15-1 OD and J1+ OS. Repeat manifest refraction was -2.50 OD and -1.75 OS. The patient was then planned for SMILE OD with a plano target. The procedure was uncomplicated with complete lenticule extraction and visualization. On postoperative day one, the patient complained of a film over the vision with an uncorrected distance visual acuity (UDVA) of 20/15-2. A well-healing superior corneal incision and a clear cap were noted without anterior chamber inflammation. The patient was started on preservative-free artificial tears QID, gatifloxacin 0.5% QID, and prednisolone acetate 1% QID. At the one-week follow-up, the patient noted improvement in the quality of vision with UDVA of 20/15-1. The slit lamp examination was unremarkable and demonstrated a clear corneal cap without ocular inflammation. The above eye drops were continued with a plan for a one-month follow-up.

However, two weeks after the initial SMILE procedure, the patient presented as a walk-in to the clinic complaining of redness, pain, and light sensitivity for one day. Slit lamp examination revealed no cornea edema, dendritic epithelial staining, or punctate epithelial erosions. The anterior segment was notable for 1-2+ cells OD without any keratic precipitates on the endothelium. No vitreous cells were noted in the vitreous. Prednisolone acetate 1% was changed to difluprednate 0.05% TID OD. The patient had improvement in symptoms with a decrease in anterior chamber inflammation to rare cells.

The patient returned to subsequent follow-ups with persistent rare cell and new-onset headaches rated 8/10 in severity. Visual acuity remained stable at 20/15-1 with normal IOP and fundus examination.

After three months of failed resolution of intraocular inflammation, routine laboratory testing was obtained for rheumatoid factor, antineutrophil cytoplasmic antibody, peripheral antineutrophil cytoplasmic antibodies, quantiferon, rapid plasma reagin (RPR), fluorescent treponemal antibody absorption (FTA-ABS), human immunodeficiency virus (HIV), angiotensin-converting enzyme levels, Lyme titers, antinuclear antibodies, free T3/T4, and thyroid-stimulation hormone (TSH). Results revealed low TSH (<0.015) with positive RPR and titer of 1:64. Reflexive testing for treponemal FTA-ABS was also positive. The patient denied a previous history of syphilis or treatment in the past. She was diagnosed with ocular syphilis and was referred to infectious disease for treatment. The patient received a two-week course of intravenous penicillin G.

The patient returned for additional follow-up without subjective complaints and a stable UDVA of 20/15-. The patient demonstrated trace cells and was started on a very slow taper of difluprednate for several months. Around six months postoperatively, the patient transitioned to prednisolone tapper with only rare cells documented in the anterior chamber. By one year, all anterior chamber cells had resolved, and the patient was tapered off of prednisolone. At the two-year follow-up, the patient has no visual complaints with visual acuity of 20/15-1 OD and J1+ OS. Resolution of anterior chamber cells persisted with the patient completely off of topical steroids.

## Discussion

The incidence of anterior uveitis after LASIK is rare. In a study of 18,488 eyes, only 18 patients (0.18%) developed uveitis after LASIK [5]. Patients were followed for an average of 36 months, with a mean ablation depth of 37.47 μm, ranging from 12-98 μm [5]. Symptoms occurred on average 21 days postoperatively or five days after withdrawal of topical steroids [5]. No clear etiology was found in 83% of the patients. Only three out of 18 patients were human leukocyte antigen B27 positive, suggesting alternative mechanisms including disruption of the blood-aqueous barrier secondary to uveal trauma. Suarez et al. hypothesized that sudden compression and decompression forces during suction resulted in disruption of the normal anterior chamber physiology. As a result, alteration in chemical inflammatory mediators, including pro-inflammatory and anti-inflammatory cytokines, created an inflammatory environment leading to clinically significant aqueous cell and flare.

The improvement in femtosecond docking platforms has led to a significant decrease in changes in IOP. Among the current femtosecond platforms used to generate LASIK flaps, the VisuMax (Carl Zeiss Meditec) demonstrates the lowest spike in IOP, with pressures ranging from 65 ± 20 mmHg compared to the Intra-Lase (Abbott Medical Optics) at 135 ± 16 mmHg, Femtec (Technolas Perfect Vision) at 205 ± 32 mmHg, and the Femto LDV (Ziemer Ophthalmic Systems AG) at 184 ± 28 mmHg [6].

Our patient underwent docking with the VisuMax femtosecond platform. Thus, it is unlikely that high spikes of IOP occurred causing damage to the uveal tissue and resulting in persistent inflammation after surgery. In our experience, most patients cannot tell when docking even occurs because the suction is very gentle.

Nevertheless, patients with other systemic diseases such as collagen vascular diseases, diabetes, and uncontrolled allergic or atopic disease may be associated with more postoperative inflammation and a longer healing course. However, this topic is controversial as patients with well-controlled and mild disease without ocular involvement or multidrug regimens may be suitable candidates for laser refractive surgery [7].

Further debate exists about performing refractive surgery on patients with a previous history of ocular infection. Ocular involvement from herpes simplex virus (HSV) or herpes zoster ophthalmicus (HZO) may be a relative contraindication for some surgeons. However, a retrospective study examining 49 eyes with a history of ocular herpes (HSV keratitis, 28 eyes; HSV eyelid lesions, 17 eyes; HZO, 4 eyes) found no ocular reactivation in all eyes after LASIK within 28 months [8]. No refractory postoperative inflammation or uveitis developed over a two-year period [8]. Herpetic disease was inactive at the time of surgery in all eyes for more than one year, along with the absence of stromal disease and abnormal corneal sensitivity [8]. Perioperative systemic antiviral prophylaxis was also used in all patients with a history of HSV keratitis [8]. In our patient, an underlying herpetic infection was considered. However, the patient denied a previous history of herpes or ocular infection. Although our patient developed persistent anterior chamber inflammation, no signs of dendritic lesions, keratic precipitates, endothelial edema, or elevated intraocular pressure were present, typical of a possible herpetic diagnosis.

Alternatively, other infectious etiologies were considered. In our patient population, a high level of HIV and syphilis is present. Louisiana is ranked fourth in the nation for HIV case rates (15.6 per 100,000 population) and 12th in the nation for primary and secondary syphilis case rates (15.1 per 100,000 population) according to the 2020 Centers for Disease Control and Prevention surveillance report.

Manifestations of ocular syphilis are very diverse as syphilis has been documented to affect almost every structure of the eye in both the early and late stages of syphilis in patients with or without HIV [9,10]. Symptoms may include eye pain, vision loss, floaters, photopsia, and photophobia [11]. The secondary stage may have ocular involvement such as keratitis, iris nodules, iridocyclitis, episcleritis, and scleritis [11]. Late in the secondary stage, chorioretinitis and vitritis may also develop but are more frequent in the tertiary stages [12]. Overall, the most common finding in ocular syphilis is panuveitis followed by isolated anterior uveitis [13]. The index of suspicion for syphilis should be high in any case of unexplained ocular inflammation [11]. Ocular syphilis may occur as early as six weeks after transmission and may be the only presenting feature of systemic syphilis [14].

Preoperatively, our patient was asymptomatic with no signs of previous or current ocular inflammation in either eye. Initial postoperative treatment was unremarkable until week two when the patient developed signs and symptoms of uveitis while still using 1% prednisolone. Topical steroid treatment was increased to difluprednate which improved symptoms and anterior chamber inflammation. Although the patient had symptomatic resolution while using topical steroids, a steroid taper could not be performed without developing rebound iritis. A systemic workup was regrettably delayed as the patient showed no chronic signs of uveitis (e.g., posterior synechia) and had minimal ocular inflammation. However, labs confirmed an active syphilitic infection which was treated with the ultimate resolution of ocular inflammation. Persistent ocular inflammation has also been documented in cataract surgery in a patient with latent untreated syphilis [15]. Thus, persistent cells after refractive or ocular surgery should be an alarming finding, warranting further workup to rule out underlying systemic diseases.

## Conclusions

Anterior uveitis after refractive surgery is uncommon. A majority of patients are often treated with no clear underlying etiology. The incidence of anterior uveitis of SMILE is even rarer with no documented incidence in the literature. As a result, persistent cell seen in refractive procedures including SMILE is an unusual and alarming finding, warranting further workup to rule out underlying systemic diseases including ocular syphilis.
